# The evolution toward integrated community health care for older people in Italy

**DOI:** 10.1007/s40520-025-03279-y

**Published:** 2025-12-10

**Authors:** A. Marengoni, A. Zucchelli, A. Padovani, E. Belli, L. Cajazzo, E. Burato

**Affiliations:** 1 Department of Clinical and Experimental Sciences, University of Brescia, Viale Europa 11, Brescia, 25123 IT Italy; 2 Aging Research Center, Department of Neurobiology, Care Sciences and Society, Karolinska Institutet and Stockholm University, Stockholm, Sweden; 3 Primary Care Unit, ASST Spedali Civili Brescia, Brescia, Italy; 4 Management Unit, ASST Spedali Civili Brescia, Brescia, Italy

**Keywords:** Community care, Community house, Older persons, Frailty, Primary care physician, Geriatrician

## Abstract

The progressive aging of the Italian population and the increasing prevalence of multimorbidity and frailty call for a reorganization of health and social care services. In this paper we aim to critically examine Ministerial Decree 77/2022, within Mission 6 of the National Recovery and Resilience Plan, which establishes new models and standards for community-based care and offers an unique opportunity to deliver care that is closer to people’s homes, fostering a holistic approach that combines clinical expertise, social support, and technological innovation. The reform places Community Houses and Health Districts at the core of integration between hospitals, general practitioners, and social services. The care of frail older adults relies on population stratification through validated tools, such as the Primary Care Frailty Index (PC-FI), and on the development of individualized care plans that integrate multidisciplinary interventions. Although general practitioners remain central in the health care system, geriatricians may play a pivotal role in multidimensional assessment, pharmacological management, health promotion, and the coordination of care teams. Telemedicine and digital tools support continuity of care and ensure traceability of clinical processes. Indicators of accessibility, equity, appropriateness, acceptability, and effectiveness are proposed, including the use of Patient-Reported Outcome and Experience Measures proposed for the evaluation of its impact. The reform’s success will depend on overcoming methodological and operational barriers, reducing regional disparities, and ensuring that clinical expertise, social support, and technological innovation deliver measurable benefits for frail older adults.

## Background

The progressive aging of the worldwide population has led to growing interest in new healthcare policies aimed at improving the management of individuals affected by chronic diseases and characterized by high clinical, social, and care complexity [1]. Demographic data are very clear: it has been estimated that by 2045, over one-third of the Italian population will be aged 65 or older [2]. Further, aging is associated with a significant accumulation of chronic-degenerative diseases and syndromes, resulting in a progressive increase in the prevalence of multimorbidity [3]. These demographic and epidemiological transitions should be accompanied by a reorganization of the health and social care systems, so far oriented towards the management of individual diseases and acute conditions with the consequence of inappropriate emergency room admissions and hospitalizations, especially in older people [4]. Another significant factor is the association between multimorbidity, poverty, and low educational attainment; chronic diseases tend to manifest earlier and accumulate more rapidly among populations with lower socioeconomic status and difficult access to health care [5]. Being multimorbid is undoubtedly an indicator of healthcare needs, but it cannot be used in isolation for population risk stratification and identification of needs [6]. A more comprehensive index is required, one that also captures functional and social domains (i.e., frailty) [7], as well as the extent of care needs, the frequency of healthcare utilization, and the degree of care coordination (i.e., complexity) [8].

In response to these desires, in recent years, numerous healthcare professionals and policymakers have proposed to shift the focus from symptoms and specific diseases to the overall health of the individuals within their social community context. Regarding the organization of care pathways, the guidelines on multimorbidity issued by the Italian Institute of Health (Istituto Superiore di Sanità) recommend the following principles [1]:

1. Improve coordination and collaboration between healthcare and social care professionals, as well as between hospitals and community-based services, and promote and integrate continuity of care.
2. Develop and utilize effective technologies and systems for the sharing of health and social care information and for the implementation of telemedicine and teleconsultation.
3. Promote education and professional training on the topics of multimorbidity, frailty, and preventive measures.

These same principles are embedded in Mission 6 of the National Recovery and Resilience Plan (PNRR) which seeks to reform community healthcare in Italy. The reforms of Mission 6 aim to promote people’s health starting from a multidimensional assessment of their needs and a comprehensive approach to their care, making use of telemedicine and digitalization — tools that enable a new management of services and of the healthcare system as a whole. The reform promoted under Mission 6 of the PNRR focuses on providing care to frail individuals within the community through local and home-based healthcare and social care services. The reform outlined in the PNRR was formalized with the publication of the DM of May 23, 2022, No. 77 [9] — Regulation defining models and standards for the development of community-based care within the National Health Service — marking its complementarity with DM 70/2015, which defined hospital care standards. With this reform, for the first time, standards for community-based care are established — just as they already exist for hospital care — and all Italian Regions are required to comply with them. DM 77 and the investments from the PNRR outline the place and responsibilities of health community care; what needs to be done now is to translate regulations and investments into clinical and organizational procedures. At the core of the reform is the Health District constituting an organizational and functional unit with a strategic role in the governance and coordination of the healthcare and social-health service network. It facilitates the integration of healthcare with the social services provided by local authorities, in coordination with the Assembly of Mayors, and in accordance with assessments of population health and social needs. The District is responsible for service planning based on population needs assessments and resource availability. It ensures the provision of healthcare and social-care services either directly or through external entities and guarantees equitable access for citizens. Furthermore, it continuously monitors the quality and performance of the services delivered. By Decree No. 278 dated March 30, 2022, ASST (Azienda Socio-Sanitaria Territoriale) Spedali Civili, one of the biggest hospitals in Northern Italy with also a community catchment area, shaped four Health Districts within the Brescia County, named Brescia, Brescia EAST, Brescia WEST, and VALLE TROMPIA populated by over 500,000 people of whom 12% are over 75 years of age.

The aim of this article is to critically examine the organizational and operational framework introduced by Ministerial Decree 77/2022 for community-based care in Italy, with reference to older adults, by analyzing the role of the geriatrician, the use of frailty and complexity stratification tools, multidisciplinary integration, and digital technologies.

### Community houses

Within each Health District the “Community Houses” (“Case della Comunità”) play a fundamental role by providing proximity healthcare services and reducing pressure on hospitals. The Community House is designed as an integrated, multidisciplinary organizational model operated by multidisciplinary teams, representing the ideal setting for delivering primary healthcare interventions and promoting social integration. Ministerial Decree 77/2022 defined the standards for the Community House [9]:

- at least one hub Community House for every 40,000–50,000 inhabitants.
- spoke Community Houses run by General Practitioners and Pediatricians, taking into account the orographic and demographic characteristics of the territory to ensure service capillarity and greater equity of access, especially in internal and rural areas.

Community Houses may represent an ideal setting for the care and support of older individuals for several reasons: community-based care allows services to be delivered near home, reducing barriers to access; unplanned hospitalization due to poor monitoring of chronic conditions or social needs may be reduced; early detection of mobility limitations and rehabilitation need may be ensured; collaboration between primary care physicians, nurses, social workers, and specialists becomes holistic. Older patient stratification by primary care physicians before accessing Community Houses is recommended and should be guided by a validated tool capable of identifying those most likely to benefit from a community-based model of care. Frailty is a state of increased vulnerability to stressors, caused by the age-related decline in physiological reserve across multiple organs and systems [10]. DM 77 emphasizes the central role of addressing frailty across the population and its early identification through validated tools. Among the various metrics currently available for assessing health in the older population, frailty is the one that best meets the criteria of multidimensionality and ease of assessment—both necessary conditions for a tool to be considered valid and applicable on a large scale. A frailty index has been suggested to identify people with multimorbidity at risk of unplanned hospitalization and with limited life expectancy [1]. Fortunately, in line with developments in other European countries such as the United Kingdom and Ireland [11], Italy has also embarked on a path of raising awareness and developing modern tools for large-scale frailty assessment developed within General Practice. Frailty is assessed using a previously validated frailty index (primary care frailty index, PC-FI), applied to a sample of 440,000 general practice patients aged 60 years and older, representative of the Italian population within the same age group [12]. Population stratification according to this index reveals that in Italy, 35.5% of individuals over 60 exhibit mild frailty, 14.4% moderate frailty, and 6.5% severe frailty [13]. In a recent survey conducted by Italia Longeva, which “mapped” frailty among individuals over 60 across Italy, the province of Brescia resulted among the most “frail” areas in Northern Italy [13]. The prevalence of severe frailty measured in primary care is around 8% in older adults living in Brescia; considering that overall, they are approximately 120,500 (ranging from 20.8 to 25.1% of the population of the four districts), the number of older people with severe frailty is around 9600. The frailty index is a useful tool to identify individual’s biological and functional decline and implicate a higher risk of adverse health outcomes such as hospitalization, disability, and mortality. However, the presence of frailty is not an exhaustive measure of complexity as the latter also implies the difficulty of managing care due to multiple interacting factors. In fact, the “complex” patient is not defined merely by the sum of individual organ-specific deficits but rather represents a unique entity who must be cared for holistically, addressing all health needs and being supported throughout the entire continuum of care, including acute phases, exacerbations, chronic stages, and end-of-life care—without neglecting the patient’s social and family context. Complexity is certainly not an on-off condition; rather, it results from a dynamic interplay of multiple, often interrelated, and evolving factors [14]. The complexity of older age is both widespread and difficult to capture: this reform offers the opportunity to investigate new approaches to assess complexity, moving beyond the simple aggregation of existing indicators. For example, the INTERMED interview is a tool that reflects a biopsychosocial approach to the integrative assessment of health care needs of patients [15]. The assessment method is based on a semistructured interview that classifies the information into the four domains of a patient’s biological, psychological, social, and health care-related characteristics. The questions and ratings in each domain are related to a time axis that is divided into past, present, and future. The main goal of INTERMED is the identification of patients with complex health care needs who need interdisciplinary care. A specific version adapted to older patients has been developed, the INTERMED for the Elderly (IM-E) [16].

Following the assessment of patient frailty and overall care complexity, outcomes are primarily determined not by an individual specialist, but by the quality of coordinated, multidisciplinary collaboration. The Community Home provides a Single Access Point where citizens can receive initial reception and orientation, integrated needs assessment, and activation of both healthcare and social services. Healthcare professionals and social workers jointly manage the citizen’s entry pathway. The primary care physician maintains clinical leadership, supported by relevant specialists. Community nurses play a pivotal role in ensuring continuity of care, promoting therapeutic education, and supporting treatment adherence. Municipal social workers are part of the team, participate in case meetings, and contribute to individualized care planning, especially for complex situations (e.g., social vulnerability). Within this integrated framework, the individualized care plan (PAI, Piano di Assistenza Individuale) constitutes the key document, delineating objectives, assigned responsibilities, frequency of clinical interactions, and measurable monitoring indicators [17].

### The role of geriatrics

Geriatricians retain specialized expertise in the evaluation and management of frail older adults with medical, neuropsychological, and social conditions. These frequently manifest through multifactorial presentations and encompass geriatric syndromes such as mobility impairment, falls, urinary incontinence, malnutrition, dementia, and delirium. A core aspect of their practice involves continuous monitoring of frailty status to identify severely frail individuals who would benefit from comprehensive multidimensional assessment [18]. The latter is essential as complexity is not just about diseases, but about the interplay of biological, psychological, and social factors. Only by evaluating the whole picture can healthcare systems deliver effective, coordinated, and patient-centered care. Beyond that, geriatricians play a pivotal role in promoting healthy ageing, preventing illness, reducing disability, and delivering evidence-based palliative and end-of-life care. Their capacity-oriented, person-centred approach ensures that clinical decision-making remains aligned with the individual needs and priorities of older adults [19]. Furthermore, geriatricians assume a guidance role in advancing the transition toward a more integrated and person-centred healthcare system, with a particular focus on preserving functional ability, strengthening preventive strategies, and supporting rehabilitation. By fostering, facilitating, and sustaining interspecialty collaboration, geriatricians define a distinctive role within Community Houses, where they contribute to multidisciplinary, person-centred interventions that bridge the gap between clinical care and social support.

### Telemedicine in the community

DM 77/2022 recognizes and integrates telemedicine as a fundamental component in the organization of community health services. It establishes that community care must include the use of telemedicine tools, such as televisit and teleassistance, in collaboration with primary care physicians and pediatricians. DM 77/2022 is an integral part of the PNRR, which allocates significant resources to the development of telemedicine to improve care for patients with chronic conditions. Finally, the decree provides for the monitoring of qualitative, structural, technological, and quantitative standards of facilities dedicated to territorial healthcare, including those related to the use of telemedicine. The digital tools must be reliable and simple; interoperability between systems that collect essential information such as diagnoses, assessment scales, Individual Care Plans (PAI), reports, and medications must be implemented and available during teleconsultations.

The criteria for the use of telemedicine will include patient/caregiver eligibility, follow-up planning, maximum response times for teleconsultations, and the traceability of care decisions.

### Action planning

The organizational model of ASST Spedali Civili provides for the presence of a physician 24 h/7 days in each Community House. Primary care physicians and on-call doctors are expected to join as well as several specialists including a geriatrician, not only to perform geriatric consultations according to established referral criteria (see below) but also to coordinate the interdisciplinary approach to older patient care. The geriatrician aims to ensure that older patients receive comprehensive responses to all their clinical, care-related, and social needs through the activation of other healthcare and social professionals, such as the family nurse, psychologist, physiotherapist, and social workers. Furthermore, the geriatrician maintains continuous communication with the primary care physician, including through teleconsultations, and coordinates the involvement of multiple specialists engaged in the care of the older patient.

Key responsibilities include:

- Pharmacological management: systematic review of the entire prescribed treatment regimen, with therapeutic reconciliation and, when appropriate, deprescribing in agreement with the primary care physician.
- Care plan adaptation: evaluation of necessary modifications in the patient’s care plan, particularly in the context of transition towards pre-terminal and terminal conditions. This involves the use of telemedicine and the activation of home-based palliative care services.

Finally, the geriatrician plays a proactive role in promoting healthy and active ageing through patient and family education on lifestyle and environmental factors. This includes providing personalized recommendations regarding physical activity, nutrition, prevention of frailty, sarcopenia and dementia, environmental modifications aimed at reducing fall risk, and adherence to medications and vaccination programs.

#### Suggested criteria for primary care physicians for referral to a geriatric consultation in community houses

Patients with PC-FI ≥ 0.21 (Primary Care Frailty Index), indicative of severe frailty, plus at least one of the following conditions which increase complexity of care:

- High social risk (loneliness, poverty, low education).
- The coexistence of multimorbidity, polypharmacy, disability, and/or cognitive decline.
- Balance and gait disturbances, recurrent falls.
- Need for therapeutic review and/or deprescribing.
- Chronic pain.
- Mood disorders, anxiety, or sleep disturbances.

### Indicators for assessing the effectiveness of a Population-Level geriatric intervention

To evaluate the effectiveness of the intervention at the population level, multiple indicators are required, as summarized below:

#### Accessibility

Ensure timely and adequate care for older adults.

- Proportion of eligible older adults successfully receiving the intervention (i.e., individuals with severe frailty)
- Time elapsed from referral to intervention delivery, reported as median and percentiles, stratified by type of service (community clinic visits, home-based assessments, consultations with general practitioners)

#### Acceptability

Assess user- and provider-reported satisfaction and perceived quality.

- Patient and caregiver satisfaction surveys evaluating perceived benefits, clarity of information, ease of access, and overall experience (use of Patient-Reported Outcome Measures - PROMs and Patient-Reported Experience Measures – PREMs) [20].
- Surveys for healthcare professionals assessing workload appropriateness, perceived quality of care delivered, interprofessional collaboration, and overall satisfaction with the intervention.

#### Appropriateness

Determine whether the care provided aligns with patients’ clinical needs and evidence-based standards (considering eligibility criteria).

- Proportion of intervention participants with ≥2 unplanned hospital admissions, emergency department visits, or falls in the preceding year, to identify high-risk individuals appropriately managed by the service

#### Equity

Ensure fair and consistent access to healthcare services regardless of socioeconomic or geographic factors.

1. Intervention uptake rates stratified by geographic area, age categories, and socioeconomic indicators (e.g., ISEE or equivalent measures of economic vulnerability).

#### Effectiveness

Evaluate the impact of the intervention on relevant health outcomes.

- Rates of emergency department visits, unplanned hospital admissions, and unplanned outpatient specialist visits during and within one year of intervention initiation.

## Conclusions

The territorial healthcare reform introduced by Ministerial Decree 77 (DM77) represents a necessary response to ongoing demographic and epidemiological transitions. The evolution of community care for older people represents a new way of connecting the existing levels of assistance. Similar community care models have been developed in other European countries such as Netherlands (NL). In the latter, the evolution of integrated community care happened over decades: the current mix comes from phased legislative reforms and provider innovation — slower, incremental, and locally variable. In Italy DM 77 sets numeric planning targets (e.g., Community Houses coverage metrics and arrangements) and the PNRR gives fixed timelines/resources for rapid rollout. In NL general practitioners (GPs) are central gatekeepers to the health system and they closely collaborate with district/community nursing. In Italy GPs are also first contacts for patients, but referral systems can be less strict than in NL. Both systems emphasize multidisciplinary working, locality (neighborhood in The Netherlands – Community House in Italy), and continuity of care. Municipalities collaborate with community nursing and general practitioners in neighborhood teams in NL and in the Community Houses in Italy.

To improve the chances of success of the reform, the actions to be implemented include: stratification of the population by frailty and levels of complexity which allows for a correct understanding of needs; interdisciplinary teams which enable more coordinated and effective actions; telemedicine and system interoperability to make decisions and results traceable. With a clear set of indicators and an active district governance, the model becomes feasible and assessable. Given that older adults represent the population most affected by multimorbidity and frailty as well as complex care needs, the geriatrician assumes a pivotal role, both as a direct provider of patient care and as a key collaborator of the primary care physician.

## Data Availability

No datasets were generated or analysed during the current study.
